# Cytoplasmic PPARγ Significantly Correlates With P53 Immunohistochemical Expression and Tumor Size in Localized Tenosynovial Giant Cell Tumor

**DOI:** 10.7759/cureus.60377

**Published:** 2024-05-15

**Authors:** Fatma Alzahraa A Elkhamisy, Elshaimaa A Aboelkomsan, Marwa K Sallam, Ahmed N Eesa

**Affiliations:** 1 Pathology Department, Faculty of Medicine, Helwan University, Cairo, EGY; 2 Pathology Department, School of Medicine, Newgiza University, Giza, EGY; 3 Medical Microbiology and Immunology Department, Faculty of Medicine, Cairo University, Giza, EGY; 4 Pathology Department, Faculty of Medicine, Cairo University, Giza, EGY

**Keywords:** immunohistochemistry (ihc), soft tissue, tenosynovial giant cell tumor, p53, ppar, multinucleated giant cells, joints, histiocytes, foamy macrophages, inflammatory tumor

## Abstract

Background: Tenosynovial giant cell tumor (TGCT) is a monoarticular fibrohistiocytic benign or locally aggressive soft tissue tumor that originates from the synovium of joints, bursae, and tendon sheaths. It has an inflammatory neoplastic nature, with a clinical presentation ranging from pain, swelling, stiffness, and limited range of movement to joint instability and blockage. Its uncommon incidence leads to a poorly understood pathogenesis. Localized forms of TGCT (LTGCT) can cause significant morbidity, interfere with daily patient activities, and decrease the patient's quality of life in challenging cases. This study aimed to investigate the immunohistochemical expression of PPARγ (peroxisome proliferator-activated receptor gamma) and P53 in LTGCT to understand the disease better and offer potential therapeutic targets.

Methods: The study is cross-sectional, in which 27 LTGCT cases were collected from the Pathology Department, Faculty of Medicine, Cairo University, Cairo, Egypt. Solitary and multiple LTGCT cases retrieved between January 2018 and December 2022 were included, and immunohistochemically stained with anti-PPARγ and P53 antibodies. The TGCT samples were excluded if they were insufficient for sectioning, processing, and interpretation, over-fixed, had process artifacts, or were of the diffuse TGCT type. Scoring of stain expression was performed by ImageJ (National Institutes of Health, Bethesda, MD) analysis using the threshold method and was expressed in percent area/high power field. Clinicopathological correlations were analyzed.

Results: All the 27 collected LTGCT cases were located in the small joints of patients' hands. Cases with solitary LGTCTs constituted 55.6% (n = 15), while 44.4% (n = 12) had multiple LTGCTs related to one affected site/case (e.g., multiple tumors in one finger). PPARγ was expressed in the cytoplasm of mononuclear and multinucleated tumor cells and foamy histiocytes, while P53 expression was mainly in mononuclear cells' nuclei. PPARγ significantly correlated with P53 expression (r = 0.9 and P = 0.000). PPARγ (r = 0.4 and P = 0.02) and P53 (r = 0.5 and P = 0.01) were positively correlated with tumor size. Only P53 expression was positively correlated with tumor multiplicity (r = 0.4 and P = 0.03). Using the receiver operating characteristic curve test, the P53 cutoff score detecting the multiplicity of TGCTs was ≥20.5%, with a 75% sensitivity and 80% specificity.

Conclusion: PPARγ and P53 have a significant role in LTGCT growth, while P53 plays a role in tumor multiplicity. They can be possible targets in LTGCTs unfit for excision.

## Introduction

Tenosynovial giant cell tumor (TGCT), formerly known as giant cell tumor of the tendon sheath and pigmented villonodular synovitis (PVNS), is a monoarticular benign or locally aggressive fibrohistiocytic soft tissue tumor that originates from the synovium of joints, bursae, and tendon sheaths [1]. Its clinical presentation is indistinguishable from many other joint diseases and ranges from pain, swelling, stiffness, and limited range of movement to joint instability and blockage [2]. It often causes morbidity, interferes with daily patient activities, and decreases the patient's quality of life [3,4]. Although many reports described its rare incidence in the literature [2], some epidemiologic studies reported a higher tumor incidence in some countries [5]. TGCT can present as a localized well-delineated form (LTGCT) with a higher incidence than the diffuse form (DTGCT) of TGCT consisting of multiple, multilobulated lesions, often extending intra- and extra-articular [6]. Multiple tumors of the localized TGCT (LTGCT) type have been increasingly reported in the literature [7,8].

The tumor composition creates a highly inflammatory microenvironment, describing the tumor as an inflammatory-neoplastic disease [2]. Till now, the pathogenesis of TGCT is not fully understood [9].

The main line of treatment for TGCT is surgical excision, either by arthroscopy or open surgery [10]. However, high recurrence and morbidity are frequently reported in the challenging cases of large-sized tumors, multiple lesions, and the diffuse form, with some cases being inoperable [9,11,12].

Several trial studies to investigate the response to target systemic therapy drugs are being implemented to offer an alternative/combination therapy for challenging cases; however, the success rates are still low [9]. Understanding protein expression patterns in TGCT will help understand its pathogenesis, offer new treatment modalities, and reduce morbidity and complications.

PPARγ (peroxisome proliferator-activated receptor gamma) is a member of the nuclear receptor superfamily of transcription factors proposed to play a role in TGCT pathogenesis. It is expressed in high levels in adipose tissue and monocyte-derived macrophages and stimulates adipocyte and macrophage differentiation [13]. It regulates immunity and inflammatory response and possesses anti-tumoral effects by inhibiting tumor proliferation, invasion, and induction of differentiation and apoptosis [14]. Stimulatory peroxisome proliferator-activated receptor (PPAR)-modulating therapy for TGCT is being investigated with initial positive outcomes [15], suggesting that PPARγ suppression plays a vital role in the pathogenesis of the tumor. Despite the initial positive results of PPAR stimulation, its role in TGCT is yet to be thoroughly investigated in the literature.

P53 (protein 53) is a tumor suppressor and cell cycle regulator that has been suggested to have a role in TGCT development. P53 mutations and P53 strong expression in TGCT are associated with apoptosis defects in monocytes and giant cells and malignant transformation [2,16].

PPARγ is involved in several inflammatory joint and bone pathologies, including gouty arthritis [17], where in some of them, its action appears to be P53-mediated, as in rheumatoid arthritis [18]. Furthermore, the PPARγ-P53 association has been described in other systemic pathologies [19,20]. Nevertheless, the association between PPARγ and P53 expression was not investigated in TGCT in the published literature. Moreover, no studies investigated these markers' expression association with tumor multiplicity.

This study aimed to investigate whether there is an association between PPARγ and P53 expression in the localized form of TGCT - the more frequent type of this uncommon disease - and their association with tumor multiplicity and size. The study seeks to understand tumor pathogenesis better to help understand it, opening new insights into target proteins and therapy modulation in the tumor.

## Materials and methods

Study design

The current investigation implemented a retrospective cross-sectional study.

Case selection

Cases of LTGCTs were collected from the Pathology Department, Faculty of Medicine, Cairo University, Cairo, Egypt. Solitary and multiple LTGCT cases retrieved between January 2018 and December 2022 were included. Available clinical and pathological data such as the patient's age, sex, tumor site, size, and number of tumors were recorded from each case's file. The cases were in the form of formalin-fixed paraffin-embedded tissue blocks, from which tissue sections were retrieved on slides. The TGCT samples were excluded if they were insufficient for sectioning, processing, and interpretation, over-fixed, had process artifacts, or were of the diffuse TGCT type.

Sample size

The sample size was calculated using the PASS 15 program (PASS 15 Power Analysis and Sample Size Software, 2017, NCSS, LLC, Kaysville, UT), setting the type 1 error (α) at 0.05 and the power (1-β) at 80%. A pilot study on 10 patients showed a positive correlation between the largest tumor diameter and each of PPARγ and P53 (r = 0.536 and 0.54, respectively). Calculations based on these values yielded a sample size of 24 cases. The study included 27 cases to compensate for tissue dropouts during the processing of slides.

Histopathological evaluation

Two consultant pathologists independently confirmed the histological diagnosis of hematoxylin and eosin slides for each case. Samples were excluded if there was insufficient material, over-fixed sections, or artifacts by the process.

Immunohistochemical examination

Four μm-thick sections of tumor blocks were mounted on positively charged slides using the avidin-biotin-peroxidase complex (ABC) method for immunohistochemical staining. The staining protocols for the investigated markers were performed according to the manufacturer’s instructions. Slides were stained by anti-PPARγ (rabbit polyclonal antibodies, ABclonal, Woburn, MA; Catalogue No. A0270, dilution 1:100) and P53 (rabbit polyclonal antibodies, ABclonal; Catalogue. No. A0263, dilution 1:100) primary antibodies. Sections were incubated with the antibodies, and the reagents required for the ABC method were added (Vectastain ABC-HRP kit, Vector Laboratories, Newark, CA). Marker expression was labeled with peroxidase and colored with diaminobenzidine (DAB, produced by Sigma-Aldrich, St. Louis, MO) to detect antigen-antibody complex. All slide-processing procedures included both positive and negative controls. The positive controls used were mouse liver and colorectal carcinoma with known P53 positivity for anti-PPARγ and P53 immunohistochemistry (IHC) staining, respectively. The omission of the primary antibody was used as a negative control for non-specific staining with a secondary antibody. IHC-stained sections were examined using an Olympus microscope (BX53, Tokyo, Japan).

Scoring of IHC results was performed by determination of reaction area percent in five high-power microscopic fields (HPF, 400x) to estimate the average percentage of immunolabel-positive cells using ImageJ software for image analysis (version 1.53t, National Institutes of Health, Bethesda, MD).

The IHC-stained sections were visualized under the Olympus microscope. Automated quantitative scoring of marker expression was performed to avoid the reported shortcomings of manual scoring regarding reproducibility [21,22]. ImageJ analysis was used to score the selected foci based on automatic analysis of the color staining intensity and reaction area, giving a percentage area score/HPF; multiple photos were captured from each sample, and the images were transformed to black and white using the software. The threshold setup was used to evaluate the percentages of cells. Threshold adjustment was carried out with the removal of background signals but without the elimination of real signals. The chosen threshold was applied to all IHC photos [23,24]. Two pathologists scored the IHC-stained slides independently, and the mean of the two pathologists' scores/10 HPFs/slide was reported. The inter-scorer difference between the two pathologists' scores was statistically insignificant.

Statistical data analysis

The collected data were tabled, coded, and analyzed using IBM SPSS Statistics for Windows, version 25.0 (IBM Corp., Armonk, NY). Shapiro-Wilk's test was used to evaluate the normal distribution of continuous data. Mean, standard deviation (± SD), and range were used for parametric numerical data, while median and interquartile range (IQR) were used for non-parametric numerical data. The Mann-Whitney test (U-test) was used to assess the statistical significance of the difference of a non-parametric variable between the two study groups. Correlation analysis (using the Spearman method) was used to evaluate the strength of association between two quantitative variables. A backward multivariable linear regression model was used to determine variables affecting PPARγ and p53. The receiver operating characteristic (ROC) curve was used to evaluate the sensitivity and specificity of P53 in detecting multiple lesion tumors. A P-value < 0.05 was considered statistically significant.

Quality measures

To enhance the consistency of the reported scoring results, we followed the recommendations proposed by Meyerholz and Beck in our methodology [21]. Additionally, we used the Reporting Recommendations for Tumor Marker Prognostic Studies (REMARK) criteria to assess the quality of our publication to improve the results' comparability among biomarker studies [25].

## Results

The 27 cases of LTGCT were all present in the small joints of patients' hands. Most cases had solitary tumors (55.6%, n = 15), while 44.4% (n = 12) had multiple tumors, all found affecting the same site/patient (e.g., in one finger). Multiplicity ranged from two to four tumors/patient. Most cases (70.4%) occurred in females. As presented by the largest diameter, the size ranged between 0.8 and 4.10 cm. The median PPARγ expression score was 18.9%/HPF (IQR: 13.8-30), and for P53 was 20.3%/HPF (IQR: 10.6-41.1). The clinicopathological data of the cases are shown in Table 1.

**Table 1:** The clinicopathological data of the study group with localized tenosynovial giant cell tumor. PPARγ: peroxisome proliferator-activated receptor gamma; P53: protein 53; IQR: interquartile range; SD: standard deviation; cm: centimeter; HPF: high-power field.

Clinicopathological variable		Value
Age (years)	Mean (±SD)	30.56 (±12.80)
	Minimum - maximum	16 - 54
Sex	Male	8 (29.6%)
	Female	19 (70.4%)
Largest tumor diameter (cm)	Mean (±SD)	2.33 (±1.05)
	Minimum - maximum	0.8 - 4.1
Number of lesions	Solitary	15 (55.6%)
	Multiple	12 (44.4%)
	Two	9 (33.3%)
	Three	2 (7.4%)
	Four	1 (3.7%)
PPARγ immunohistochemical score (area %/HPF)	Mean (±SD)	22.05 (±14.56)
	Median (IQR)	18.9 (13.8 - 30.0)
	Minimum - maximum	0.64 - 53.62
P53 immunohistochemical score (area %/HPF)	Mean (±SD)	25.25 (±16.10)
	Median (IQR)	20.3 (10.6 - 41.1)
	Minimum - maximum	6.03 - 55.78

Using Spearman's correlation coefficient test, the P53 IHC score but not PPARγ significantly correlates positively with the multiplicity of tumors (r = 0.4 and P = 0.03). PPARγ and P53 were found to significantly correlate positively with each other expression (r = 0.9 and P = 0.000) and with tumor size (r = 0.4 and P = 0.02; r = 0.5 and P = 0.01, respectively). There is a statistically significant inverse correlation between age and largest diameter (r = -0.4 and P = 0.02) (Table 2).

**Table 2:** Correlations between clinicopathological findings of the cases of localized tenosynovial giant cell tumor and PPARγ & P53 immunohistochemical expression score by ImageJ analysis. PPARγ: peroxisome proliferator-activated receptor gamma; P53: protein 53; Rho: Spearman rank correlation value; P: value of significance; cm: centimeter; IHC: immunohistochemistry; HPF: high-power field.

Clinicopathological findings of localized tenosynovial giant cell tumor cases	Spearman's correlation coefficient		
	Value	PPARγ IHC score (area %/HPF)	P53 IHC score (area %/HPF)
Age (years)	Rho	-0.294	-0.433
	P	0.136	0.024
Largest diameter of the tumor (cm)	Rho	0.441	0.468
	P	0.021	0.014
Number of tumors	Rho	0.319	0.413
	P	0.104	0.032
PPARγ IHC score (area %/HPF)	Rho	1	0.932
	P	0.000	0.000

**Figure 1:**
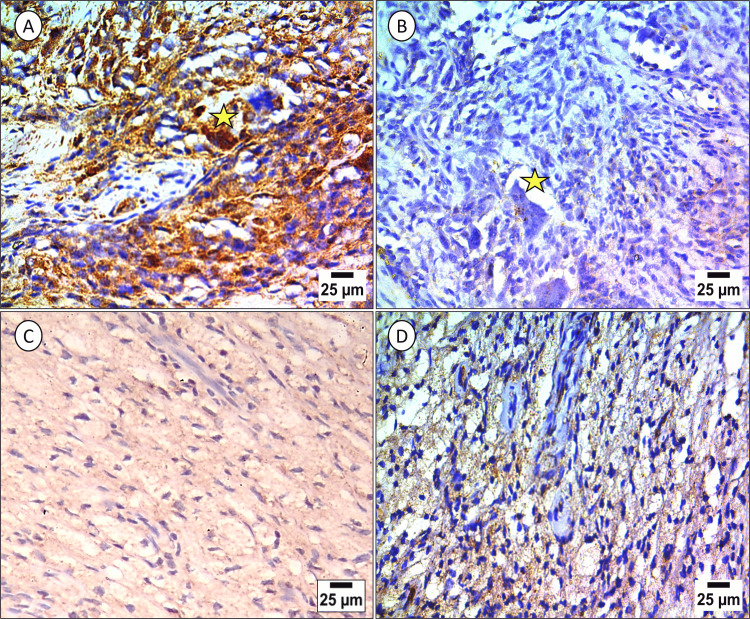
Immunohistochemical expression of PPARγ in localized tenosynovial giant cell tumor (LTGCT). Peroxisome proliferator-activated receptor gamma (PPARγ) expression in large-sized localized tenosynovial giant cell tumor (LTGCT) lesions is prominent in the cytoplasm of multinucleated giant cells (starred) and epithelioid mononuclear cells (A) compared to its low expression in the corresponding cell types of small-sized lesions (B). The foamy macrophage component shows positive granular staining in both the large (C) and small (D) lesions (original magnification, x400).

Histologically, the PPARγ IHC expression in the tumor was cytoplasmic with few scattered cells showing nucleocytoplasmic expression. Both mononuclear and multinucleated cell components expressed staining in different intensities. Expression in the multinucleated giant cells and the epithelioid-like mononuclear cells was more marked in the multiple large-sized tumors than in solitary small ones. The expression of PPARγ in the foamy macrophage component of the LTGCT was noticed regardless of the size of the tumor (Figure 1).

**Figure 2:**
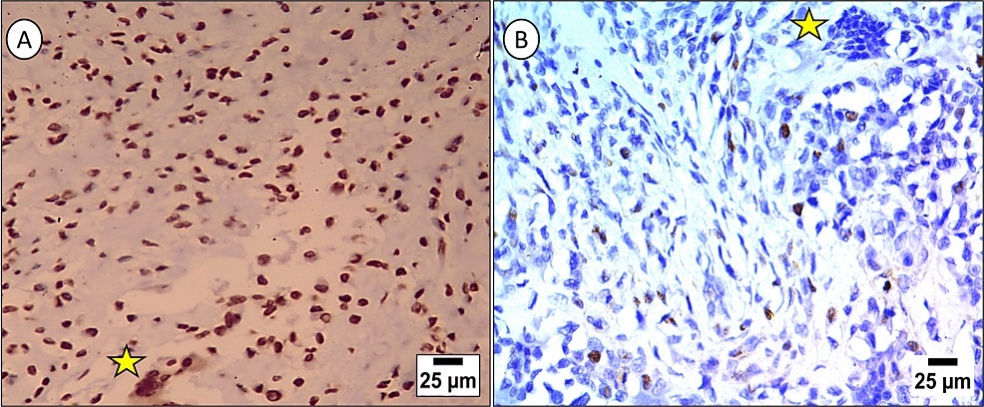
Immunohistochemical expression of P53 in localized tenosynovial giant cell tumor (LTGCT). Protein 53 (P53) nuclear expression is prominent in the mononuclear cells and some multinucleated giant cells (starred) of multiple/large-sized localized tenosynovial giant cell tumor (LTGCT) lesions (A) compared to its lower expression in the corresponding cell types of solitary/small-sized lesions (B) (original magnification, x400).

The IHC expression of P53 was nuclear with few scattered cells showing nucleocytoplasmic expression. P53 was mainly expressed in mononuclear cells compared to the multinucleated ones, which were occasionally stained (Figure 2).

Studying the relation between each sex, lesion numbers, PPARγ, and P53 immunohistochemical expression (area %/HPF) using the Mann-Whitney test showed that the multiplicity of the tumor is only significant with P53 expression (P = 0.017) (Table 3).

**Table 3:** Relationship between sex and lesion multiplicity in localized tenosynovial giant cell tumor cases, with PPARγ and P53 immunohistochemical (IHC) expression score by ImageJ analysis. * Mann-Whitney test; CI: confidence interval; SD: standard deviation; IQR: interquartile range; PPARγ: peroxisome proliferator-activated receptor gamma; P53: protein 53; IHC: immunohistochemistry; HPF: high-power field.

Localized tenosynovial giant cell tumor character		PPARγ IHC score (area %/HPF)					95.0% lower CI	95.0% upper CI	P*	P53 IHC score (area %/HPF)					95% lower CI	95% upper CI	P*
		Mean	±SD	Median	IQR*					Mean	±SD	Median	IQR*				
Sex	Male	24.59	5.96	25.5	20.1	29.2	19.61	29.57	0.24	33.42	11.16	34.8	25.4	42.8	24.1	42.7	0.05
	Female	20.98	16.97	16.5	9.2	31.9	12.80	29.16		21.81	16.84	14.3	9.2	40.6	13.6	29.9	
Multiplicity	Solitary	18.71	17.25	14.2	2.3	30.0	9.21	28.20	0.07	19.35	16.10	11.7	8.4	20.5	10.4	28.2	0.01
	Multiple	26.22	9.60	25.5	17.8	31.0	20.12	32.32		32.62	13.26	34.8	18.7	44.9	24.2	41.1	

**Figure 3:**
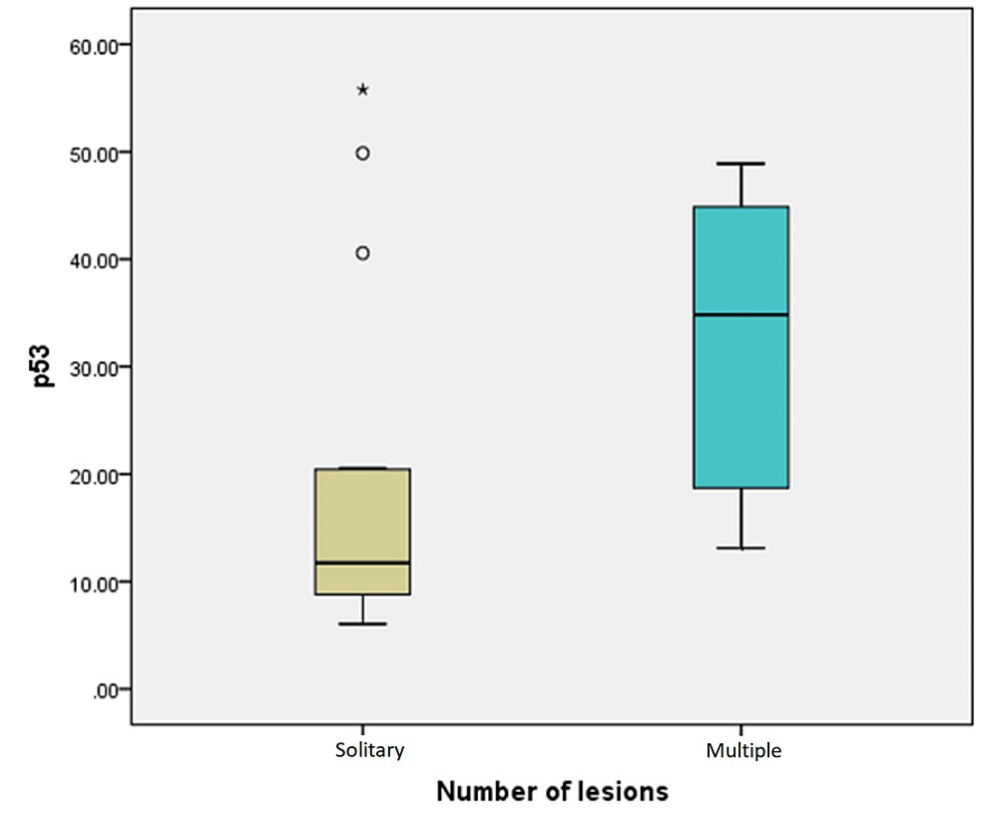
Box whisker plot showing P53 immunohistochemical expression score (in area %/HPF) in solitary & multiple localized tenosynovial giant cell tumor lesions. P53: protein 53; HPF: high-power field.

Using a linear regression model to study independent factors affecting PPARγ and P53 expression in LTGCTs showed that the tumor's largest diameter is the only significant affecting factor (P = 0.46 and P = 0.2, respectively); however, the age, sex, or multiplicity are not significant (Table 4 and Figure 3).

**Table 4:** Linear regression model to study independent factors affecting PPARγ & P53 immunohistochemical expression in localized tenosynovial giant cell tumor cases. *B: regression coefficient; CI: confidence interval; PPARγ: peroxisome proliferator-activated receptor gamma; P53: protein 53; P: the value of significance; IHC: immunohistochemistry; cm: centimeters; HPF: high-power field.

Clinicopathological criterion of the tumor	PPARγ IHC score (area %/HPF)				P53 IHC score (area %/HPF)			
	B*	P	95.0% CI for B*		B*	P	95.0% CI for B*	
			Lower bound	Upper bound			Lower bound	Upper bound
Age (years)	0.081	0.806	-0.595	0.756	0.166	0.627	-0.533	0.864
Sex	11.035	0.468	-20.031	42.100	5.936	0.705	-26.179	38.051
Largest diameter (cm)	5.338	0.046	0.097	10.579	6.727	0.021	1.085	12.369
Number of tumors	-14.142	0.176	-35.164	6.879	0.166	0.627	-0.533	0.864
Solitary versus multiple tumors	22.662	0.342	-25.789	71.113	5.936	0.705	-26.179	38.051

Using the ROC curve test, the P53 IHC score was more significant in detecting the multiplicity of TGCTs than PPARγ (P = 0.005). The cutoff point for the P53 expression score was ≥20.5%/HPF with a sensitivity of 75%, specificity of 80%, positive predictive value (PPV) of 75%, negative predictive value (NPV) of 80%, and area under the curve (AUC) (confidence interval) of 0.772 (0.58-0.96).

## Discussion

TGCT is a rare inflammatory arthritis with neoplastic features [2]. It consists of two populations of mononuclear cells within a hyalinized stroma: small histiocyte-like cells with round to oval nuclei, frequently making up the majority of the mononuclear cellular component, and larger epithelioid cell component with lots of cytoplasm and larger nuclei [1]. In addition, the tumor contains foamy macrophages, inflammatory cells, siderophages, and osteoclast-like multinucleated large cells in varying amounts [9]. These cells create an inflammatory microenvironment within the affected joints [2].

The TGCT has unclear definitive pathogenesis [9]. It has two types: the localized type, which is more common than the diffuse type [1]. It has a considerable morbidity, especially with multiple, large, or diffuse lesions [3,11]. Understanding the IHC pattern expression in tumors can open new opportunities to design personalized treatment plans and implement precision medicine [26]. Our study is the first to investigate the correlation between PPARγ and P53 expression in LTGCT and showed that they significantly positively correlate with each other expression and LTGCT size. P53 but not PPARγ expression significantly correlates positively with tumor multiplicity.

In our study, PPARγ and P53 expression varied between different cell types. PPARγ was expressed in multinucleated giant cells of larger tumors compared to small-sized ones. On the other hand, P53 expression was dominant in the mononuclear cell component, while the multinucleated cells showed occasional expression. TGCT is a heterogenous tumor with multiple cell origins. The mononuclear cells have phenotypic characteristics associated with a monocyte/macrophage lineage, while the multinucleated giant cells exhibit an osteoclast-like phenotype [27]. We suggest that the varying differential expression of PPARγ and P53 in TGCT cellular components and their interaction impacts tumor growth.

The PPAR family members are ligand-activated transcription factors with three isotypes, PPAR α, γ, and δ. PPARγ plays a crucial role in glucose metabolism, fatty acid oxidation, cell cycle regulation, adipocyte differentiation, lipid storage, and inflammation [13]. The exact role of PPARγ in tumors as oncogenic versus tumor suppressive remains controversial [23]. PPARγ association with neoplastic pathogenesis has recently been increasingly recognized. PPARγ is involved in the metabolic reprogramming of neoplastic cells, tumor cell-associated secretions, tumor microenvironment, adaptations, and the host immune response to tumors [13]. Besides, as fatty acid metabolism is vital for neoplastic development and PPARγ is crucial for fatty acid metabolism, higher PPARγ signaling is linked to neoplastic growth [28]. High fatty acid metabolism provides neoplastic cells with sufficient membranes and energy to support their growth [29]. Moreover, fatty acid oxidation has been reported as a necessary factor for macrophage activation [16]. Higher PPARγ signaling is linked to macrophage polarization in the tumor microenvironment, especially the M2 type [28].

High PPARγ expression increases cell proliferation in other neoplastic disorders [30]. Our study implies that higher PPARγ is consistent with higher tumor growth reflected by the larger size. Contrary to our results, another study reported that induced PPARγ upregulation is associated with wide TGCT tumor necrosis [15]. The differences in reported results can be explained by the varying role of PPARγ depending on varying tumors, individual characters, and PPARγ concentration [23]. Moreover, although PPARγ is a nuclear receptor transcription factor, different subcellular locations of IHC expression were described in the literature and were associated with different neoplastic features and prognoses [13,23,30]. PPARγ expression was found to be cytoplasmic in all cases of our study. Recent studies have described that cytoplasmic PPARγ expression is linked to unique tumor criteria that are tissue-dependent [23,30] and that it is inversely related to nuclear expression [30]; however, the exact mechanism and significance are not fully understood [23].

In our study, higher P53 expression significantly correlates to larger tumor size, multiplicity, and PPARγ expression. This is consistent with the literature in which P53's strong expression in TGCT was reported to be associated with defects of apoptosis in monocytes and giant cells and with malignant transformation [2,16]. The gene expression profile of TGCT is consistent with apoptosis resistance, inflammation, and matrix degradation, leading to ongoing proliferation and joint destruction [9].

Although the PPARγ and P53 association was not investigated in TGCT before, their interplay was reported in other diseases in the literature [18-20]. Some studies showed that ligand activation of PPARγ in monocytes/macrophages in other disease types inhibits inflammatory mediator and cytokine production [14]. Furthermore, specific PPARγ ligands can induce growth inhibition and apoptosis in certain neoplasms and inflammatory diseases through P53-dependent mechanisms [9]. We propose that the difference in our results can be attributed to the documented variance in the PPARγ role, which is tumor-, individual-, and concentration-dependent [23].

More research is needed to investigate the interaction between PPARγ, P53, and the downstream inflammatory molecules in large-sized and multiple TGCTs. Also, research to investigate the association between drug treatments received by the patients (e.g., antidiabetic medications) and PPARγ expression in LTGCT is suggested. The limitations of our study include the small number of cases attributed to the disease's rarity, besides being a single institution study. Further multicenter research with a higher number of cases is recommended. The study's strengths include, besides being the first to investigate PPARγ and P53 correlation in the tumor and their relation to tumor multiplicity, using quantitative scoring through image analysis to eliminate subjectivity, producing a higher dynamic range of data for better analysis compared to the visual and quantitative categorical scores [22].

## Conclusions

PPARγ and P53 expression is significantly associated with larger LTGCT size, and P53 is associated with the multiplicity of tumors. PPARγ significantly correlates with P53 expression in LTGCTs. These results can help understand the pathogenesis of the challenging cases of multiple and large TGCTS, which cause higher morbidity and help in personalized treatment and precision medicine studies. Targeting either PPARγ and P53 or P53 alone might be of value in treating large and multiple LTGCTs, respectively.
